# Ciliated Hepatic Foregut Cyst: Two Case Reports in Children and Review of the Literature

**DOI:** 10.1155/2013/372017

**Published:** 2013-10-03

**Authors:** Maliheh Khoddami, Maryam Kazemi Aghdam, Azadeh Alvandimanesh

**Affiliations:** ^1^Department of Pathology, Pediatric Pathology Research Center, Mofid Pediatric Medical Center, Shahid Beheshti University of Medical Sciences, Tehran 15468-15514, Iran; ^2^Pediatric Pathology Research Center, Mofid Pediatric Medical Center, Shahid Beheshti University of Medical Sciences, Tehran 15468-15514, Iran

## Abstract

Ciliated hepatic foregut cyst (CHFC) is a rare lesion which originates from detached hepatic diverticulum or from detachment and migration of buds from the esophageal and bronchial regions of the foregut which subsequently get entrapped by the liver during the early embryonic development of the foregut. CHFCs are mostly seen in adults and are rarely reported in children, with only about 10 cases reported in this age group. Hereby, we present two cases of CHFC in two 3.5-year-old boys; one of them had cystic lesion at medial segment of left lobe of liver (common site), and in the other one it was located at right lobe of liver (less common site). Histologically, both cysts had four layers composed of inner ciliated, pseudostratified, columnar epithelium; subepithelial connective tissue; smooth muscle layer; and an outer fibrous layer.

## 1. Introduction

Ciliated hepatic foregut cyst (CHFC), a rare foregut developmental malformation, usually presents as a solitary, unilocular, or occasionally multilocular cyst in the liver [1–9]. This congenital lesion is rare and is mostly diagnosed in adults, with estimated 100 cases reported in the world literature, many of them in Japan [1–3, 6–8, 10–12]. Only 10 cases were reported in children in our review of English medical literature [1, 4–6, 13–15]. Patients are usually asymptomatic and they are mostly detected incidentally on radiologic imaging or during surgical exploration. However, patients may present with portal hypertension and obstructive jaundice, and they may even present with malignancy, which happens in 3% of cases [2, 6, 9, 11, 16–18]. Vague right upper quadrant pain, nausea, and vomiting are the most common symptoms [19]. Most CHFCs are well-delineated anechoic or slightly hypoechoic unilocular cysts on ultrasonography (US) and they are hypoattenuating or isoattenuating relative to liver parenchyma on enhanced computed tomographic scan (CT); however, radiology alone is not diagnostic [8]. Only histological features with a four-layered cyst wall, which consists of an inner ciliated, pseudostratified, columnar epithelium followed by subepithelial connective tissue, smooth muscle layer, and an outer layer fibrous tissue [1, 2, 4–7, 9, 10, 12, 15, 19], are characteristic of this lesion. We present two cases of CHFC in children because of their extreme rarity. These are the first pediatric cases to be reported from Iran.

## 2. Case 1

**Figure 1:**
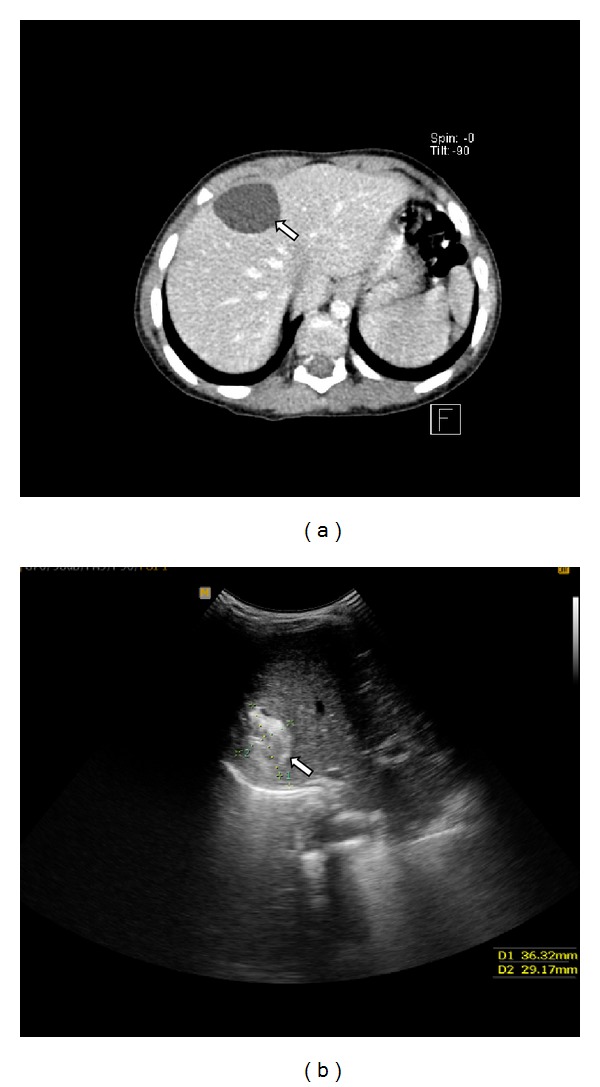
(a) CT shows a simple 3.7 × 2.8 cm subcapsular liver cyst in median segment of left lobe (left arrow). (b) US shows a 3.6 × 3.2 cm heterogeneous lesion in right lobe of liver (left arrow).

A 3.5-year-old boy presented with right upper quadrant abdominal pain. At the ninth month of gestation, a liver cyst was detected by US (no photographs were available). No further investigation was done after birth. Physical examination was normal. Laboratory tests showed mild microcytic anemia (Hb: 10.7 gr/dL, normal range: 11–14; MCV: 71.9 FL, normal range: 73–85); the other tests, including liver function tests, were within normal limits. Ultrasonography and CT showed a simple 3.7 × 2.8 cm subcapsular liver cyst in median segment of left lobe with no enhancement at CT (Figure 1(a)). He underwent laparotomy and cyst removal. The specimen consisted of a previously opened cyst with smooth external and internal surfaces, measuring 2.3 × 2 cm in diameter; wall thickness was up to 0.1 cm.

## 3. Case 2

A 3.5-year-old boy was referred to the hospital with constipation. Physical examination was normal. A hepatic cyst was detected by US. Hematologic test showed mild normocytic anemia (Hb: 10.9 gr/dL, normal range: 11–14; MCV: 81 FL, normal range: 73–85). Serum electrolytes and liver function test were within normal limits. Ultrasonography showed a heterogeneous lesion, measuring 3.6 × 3.2 cm in right lobe of liver (Figure 1(b)), and CT showed a hypodense cystic lesion without enhancement. The cyst was resected with clinical and radiological diagnosis of hydatid cyst and the specimen consisted of a previously opened cyst with rough brown external and internal surfaces, measuring 3.5 × 2.5 cm in diameter and up to 0.5 cm in wall thickness.

**Figure 2:**
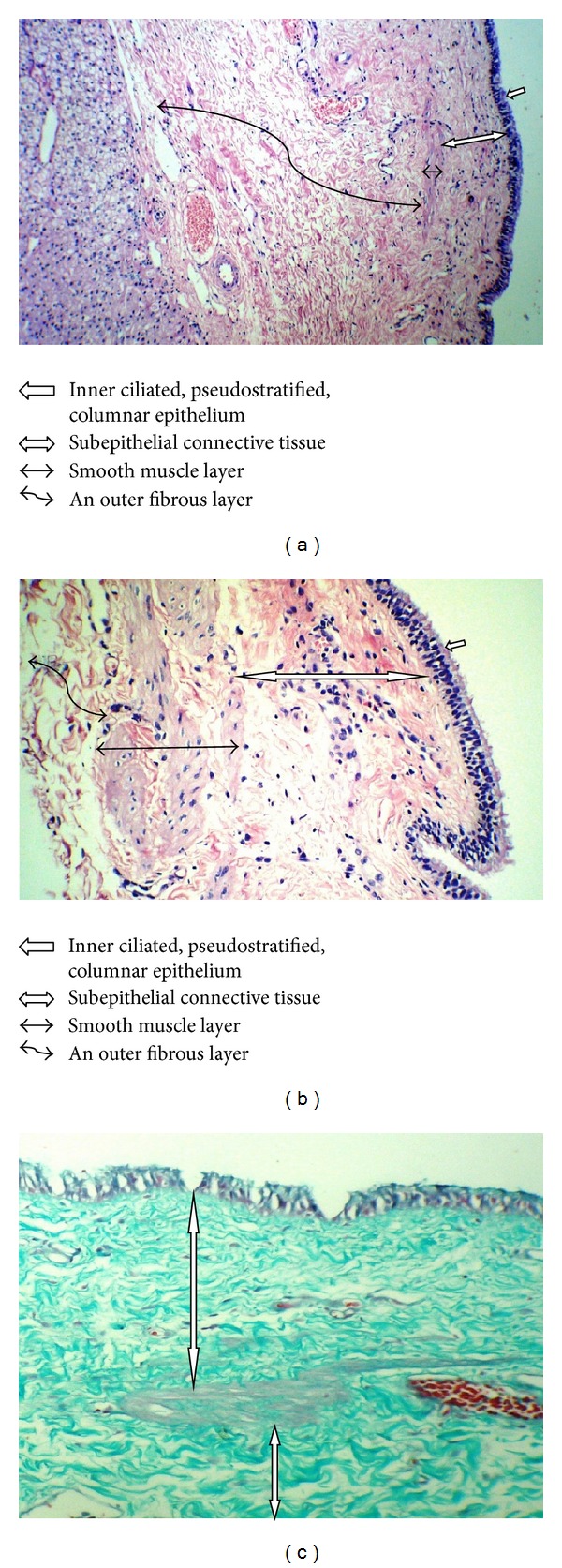
(a) Liver tissue and cyst wall composed of four typical layers (H&E ×250). (b) Four layers of cyst higher magnification (H&E ×400). (c) The fibrous layers of the cyst are stained green by Masson trichrome stain (*⇕*) (Masson trichrome stain ×400).

At histological examination, both cysts had four typical layers of ciliated hepatic foregut cyst, composed of inner ciliated, pseudostratified, columnar epithelium; subepithelial connective tissue; smooth muscle layer; and an outer fibrous layer (Figures 2(a) and 2(b)). Foci of squamous metaplasia without atypia were detected in case 2. The fibrous layers are stained green with Masson trichrome stain (Figure 2(c)). Postoperation course was uneventful in both patients.

## 4. Discussion

The incidence of hepatic cysts is approximately 5% in general population; they are classified into parasitic and nonparasitic cysts. One of the uncommon types of liver cyst is CHFC, which arises from the embryonic foregut. It is believed that it originates from a detached hepatic diverticulum or from detached and migrated foregut buds, entrapped in liver at the early embryonic development of the foregut [20]. Because CHFCs are usually asymptomatic, it is difficult to determine their prevalence. They are usually found in the medial segment of the left lobe beneath the hepatic capsule, but can rarely be found in right lobe [4–7, 9, 13, 14, 19]. One of our cases was located in medial segment of left lobe and the other one was in right lobe.

These lesions are extremely rare in children and only 10 cases have been reported (Table 1) [1, 4–6, 12–15]. In pediatric population, including this report, five out of 12 cases (41.7%) were diagnosed antenatally and seven (58.3%) were detected postnatally. Both sexes were equally affected. Seventy-five percent of cases (9 patients) were diagnosed before age 5 (including antenatally diagnosed cases) and the remaining 25% (3 cases) were detected between the age 10 and 14 years. Nine patients (75%) were asymptomatic and 25% had symptoms related to the cyst. The maximum size of the cyst was between 0.6 and 11 cm. One of our cases was detected at prenatal sonography and was asymptomatic until 3.5 years of age; the second one had no symptoms related to his hepatic cyst. Both of our cases were male and the cysts were 3.5 and 3.7 cm in maximum diameter.

The mean age at diagnosis is 50 years (3 months to 82 years), with a slight male predominance [6, 10, 19]. They are usually unilocular cysts [1, 3, 9, 19] as they were in our cases. The differential diagnoses include simple cysts, parasitic cysts, complex biliary cyst, intrahepatic choledochal cyst, cystic mesenchymal hamartoma, and cystic malignant tumors [3, 6, 19, 21]. The typical microscopic finding of four layers (the inner ciliated, pseudostratified, columnar epithelium; subepithelial connective tissue; smooth muscle layer; and an outer fibrous layer at microscopic examination) excludes the other diagnoses [1, 2, 4–7, 9, 10, 12, 15, 19]. Both of our cases had similar histological findings; one case also had focal squamous metaplasia. Presence of squamous metaplasia is also reported in other studies [1, 4] and is considered important because of possibility of transformation into squamous cell carcinoma, as reported in a few cases [1, 4, 9, 16–18].

The clinical course is usually benign; however, malignant transformation to squamous cell carcinoma with aggressive behavior has been reported in 3% of cases, all of which happened in adults between 21 and 51 years of age, supporting the idea of excision of the cyst when discovered in adults, and careful clinical followup of the patients [1, 9, 16–18]. In children, some recommend that the cyst removal should be performed after one year of age in asymptomatic cases, due to high risk of hepatic procedures [4]. However, one may argue that in asymptomatic children, it would be wise to monitor clinically and do the surgery when it is necessary. Many cases can be removed laparoscopically [19, 22]. 

Since hydatid cyst is endemic in our country, it is usually at the top of the differential diagnoses list for a liver cyst, such as case 2. Inclusion of CHFC in the differential diagnosis and performance of serological tests to exclude hydatid cyst could make laparoscopic removal more likely with less postoperative complications. We have presented two cases of CHFC in 3.5-year-old boys due to extreme rarity of this lesion in children. CHFC in children is not previously reported from Iran. 

## Figures and Tables

**Table 1:** Demographic and clinical description of the 10 pediatric and adolescent patients of CHFC.

Study	Age/gender	Size (cm)	Symptoms
Stringer et al. [15]	Prenatal/M	10 × 7.5 × 5	Yes
Betalli et al. [13]	Prenatal/F	4 × 5	No
Kim et al. [5]	3 years/M	2 × 1.5	No
Carnicer et al. [14]	5 years/F	2 × 1.3	No
Vick et al. [6]	14 years/F	6 (diameter)	Yes
Guérin et al. [4]	Prenatal/M	8.8 × 8.3 × 6.1	No
Guérin et al. [4]	Prenatal/F	2.2 × 1.2	No
Guérin et al. [4]	10 years/M	2 (diameter)	No
Guérin et al. [4]	10 years/F	0.6 (diameter)	No
Deshmukh et al. [1]	2 years/F	11 × 10 × 7	Yes
